# Extended Ultrastructural Characterization of Chordoma Cells: The Link to New Therapeutic Options

**DOI:** 10.1371/journal.pone.0114251

**Published:** 2014-12-05

**Authors:** Dagmar Kolb, Elisabeth Pritz, Bibiane Steinecker-Frohnwieser, Birgit Lohberger, Alexander Deutsch, Thomas Kroneis, Amin El-Heliebi, Gottfried Dohr, Katharina Meditz, Karin Wagner, Harald Koefeler, Gerd Leitinger, Andreas Leithner, Bernadette Liegl-Atzwanger, Dagmar Zweytick, Beate Rinner

**Affiliations:** 1 Center for Medical Research, Medical University of Graz, Graz, Austria; 2 Institute of Cell Biology, Histology & Embryology, Medical University of Graz, Graz, Austria; 3 Ludwig Boltzmann Institute for Rehabilitation of Internal Diseases, Ludwig Boltzmann Cluster for Rheumatology, Balneology and Rehabilitation, Saalfelden, Austria; 4 Department of Orthopaedic Surgery, Medical University of Graz, Graz, Austria; 5 Division of Hematology, Department of Internal Medicine, Medical University of Graz, Graz, Austria; 6 Institute of Pathology, Medical University of Graz, Graz, Austria; 7 Biophysics Division, Institute of Molecular Biosciences, University of Graz, Graz, Austria; Simon Fraser University, Canada

## Abstract

Chordomas are rare bone tumors, developed from the notochord and largely resistant to chemotherapy. A special feature of this tumor is the heterogeneity of its cells. By combining high pressure freezing (HPF) with electron tomography we were able to illustrate the connections within the cells, the cell-cell interface, and the mitochondria-associated endoplasmic reticulum membrane complex that appears to play a special role among the characteristics of chordoma. These lipid raft-like regions are responsible for lipid syntheses and for calcium signaling. Compared to other tumor cells, chordoma cells show a close connection of rough endoplasmic reticulum and mitochondria, which may influence the sphingolipid metabolism and calcium release. We quantified levels of ceramide and glycosylceramide species by the methyl tert-butyl ether extraction method and we assessed the intracellular calcium concentration with the ratiometric fluorescent dye Fura-2^AM^. Measurements of the changes in the intracellular calcium concentration revealed an increase in calcium due to the application of acetylcholine. With regard to lipid synthesis, glucosylceramide levels in the chordoma cell line were significantly higher than those in normal healthy cells. The accumulation of glycosylceramide in drug resistant cancer cells has been confirmed in many types of cancer and may also account for drug resistance in chordoma. This study aimed to provide a deep morphological description of chordoma cells, it demonstrated that HPF analysis is useful in elucidating detailed structural information. Furthermore we demonstrate how an accumulation of glycosylceramide in chordoma provides links to drug resistance and opens up the field for new research options.

## Introduction

Chordoma is a rare primary malignant bone tumor that occurs with a reported incidence of 0.08 per 100,000 people [1]–[3]. This neoplasm mainly arises in the axial skeleton distributed from the base of skull to the coccyx [1], [2], [4]. Chordomas are thought to develop from notochordal remnants in the axial skeleton [4]–[6]. They are locally destructive and are often not diagnosed until they have reached a large size [7]. Chordomas have proven to be largely resistant to conventional ionizing radiation and chemotherapy, thus treatment options are mainly restricted to surgical excision [7]–[9]. What is most impressive about chordomas, however, is their unique phenotype in tumor tissue and especially in cell culture. These tumors are morphologically composed of heterogeneous cells, ranging from smaller non-vacuolated spindly-shaped cells to large cells with prominent vacuoles; the latter are referred to as the physaliferous cells [10]. Furthermore, Eĺ-Heliebi *et al*. [11] found four candidate genes that were possibly responsible for the heterogeneity in cell development by phenotype-specific analyses of the small non-vacuolated and the large physaliferous cells within two independent chordoma cell lines. Therefore, we postulate that a detailed ultrastructural testing of the vacuoles, observed in this chordoma cell type, represents an important step towards an improved understanding of their tumor biology.

Currently, the most efficient way to obtain information on cell interactions when using electron microscopy is by combining high pressure freezing (HPF) with electron tomography. The combination of both techniques enables us to visualize the dynamic process of the cell compartments at any known time point, in three dimensions, as close as possible to the native state; furthermore, it is possible to obtain interactions with reduced artifacts (extraction, condensation of proteins, or structural distortion) which are often generated by chemical fixation. Detailed ultrastructural analysis offers valuable information on the biological behavior of chordomas.

In addition to the ultrastructural characterization of the vacuoles from chordoma, as early as 1968 Erlandson et al. [10] had already showed, an accumulation of complexes where mitochondrial membrane was tightly associated with the endoplasmic reticulum (ER), a formation termed MAM (mitochondria-associated ER membrane). This conspicuous surrounding area of most mitochondria composed by the rough ER seems to be very specific to chordoma. The main function of the MAM complex is to enable the transfer of lipids and calcium between the two organelles, and it is also involved in mitochondrial physiology and apoptosis [12], [13]. Based on the number and size of MAM complexes and due to the high number of vacuoles, we investigated the mechanisms of intracellular calcium (Ca^2+^) signaling via stimulation through acetylcholine (ACh) to promote Ca^2+^ release, and the sphingolipid metabolism in form of lipid composition. Ca^2+^ signaling plays a role in many cellular processes, however, altered expression of specific Ca^2+^_channels and pumps is a characterizing feature of some cancers [14]. Changes ascribed to sphingolipid metabolism characterize different cancers and are important for maintenance of the cancer phenotype [15]. Ceramides have been known to regulate programmed cell death.[16], [17]. Glycosylceramide synthase catalyzes the glycosylation of ceramide to glycosylceramids (GlyCer), which have been found to involve many cellular processes such as cell proliferation [18] and tumor metastasis [19], [20]. Drug resistance has also been strongly associated with GlyCer [21], [22].

Our aim was to find a new perspective on the biology of chordoma that would open up the field for completely new therapeutic approaches with regard to lipids and Ca^2+^ pathways to finally overcome thus far untreatable chordoma tumors.

## Materials and Methods

### Cell culture

MUG-Chor1 [23], U-CH-1 (ACC-748; DSMZ, Braunschweig, Germany) [24], and U-CH2 (ACC-749; DSMZ) chordoma cell line cells were cultured in IMDM/RPMI 4∶1 (Invitrogen, Karlsruhe, Germany) supplemented with 2 mM L-glutamine (Invitrogen), 10% FBS (Biochrom, Berlin, Germany), and 1% ITS (Invitrogen). U2OS (osteosarcoma; purchased from ATCC, Wesel, Germany) and human skin fibroblasts, fibro-MUG-Chor1 (established from the MUG-Chor1 chordoma patient) were cultured in DMEM, 2 mM L-glutamine and 10% FBS. All cells were kept in a 5% CO_2_ atmosphere at 37°C, periodically checked for mycoplasma by PCR and verified by short tandem repeat analysis using PowerPlex 16 System Kit (Promega, Mannheim, Germany) (data not shown).

### Chemical fixation for electron microsopy

MUG-Chor1, U-CH2, and U2OS cells were grown on an Aclar film (Gröpl, Tulln, Austria), fixed in 2.5% (wt/vol) glutaraldehyde and 2% (wt/vol) paraformaldehyde in 0.1 M phosphate buffer, pH 7.4, for 2 h, postfixed in 2% (wt/vol) osmium tetroxide for 2 h at room temperature, dehydrated in graded series of ethanol and embedded in TAAB (Agar Scientific, Essex, GB) epoxy resin.

Ultrathin sections (70 nm thick) were cut with a UC 7 Ultramicrotome (Leica Microsystems, Vienna, Austria) and stained with lead citrate for 5 min and with uranyl acetate (UAc) for 15 min. Images were taken using a Tecnai G2 20 transmission electron microscope (FEI, Eindhoven, Netherlands) with a Gatan ultrascan 1000 charge coupled device (CCD) camera (temperature −20°C; acquisition software Digital Micrograph; Gatan, Munich, Germany). Acceleration voltage was 120 kV.

### High pressure freezing with a Leica EM HPM 100 and freeze substitution

MUG-Chor1, U-CH2, and U2OS cells were grown on carbon coated sapphire discs. Fresh cells on sapphire discs within the media were loaded and frozen using 2000 bar under liquid nitrogen conditions within milliseconds.

Freezing was followed by freeze substitution in acetone by adding 2% osmium tetroxide OsO_4_ and 0.2% UAc (best cryopreservation was obtained with 2% OsO_4_ and 0.2% UAc, unpublished data Kolb *et. al*) were added at temperatures below −70°C. The water in the form of ice in the MUG-Chor1 cells was replaced by substitution media. After substitution, the samples were embedded in Epoxy resin [25].

### Serial sectioning for electron microscopy

High pressure frozen MUG-Chor1 cells were sectioned across a distance of 1.4 µm. The electron micrographs of 20 sections were merged with Adobe Photoshop CS43 (Adobe Systems Inc., San José, CA) and a 3D reconstruction of the MUG-Chor1 cells, especially of the vacuoles, was done with AMIRA® software (FEI, visualization Sciences Group).

### Electron tomography

MUG-Chor1 cells were frozen in high pressure conditions, freeze substituted, chemically fixed, cut in semithin sections (300 nm) and stained with UAc and lead citrate. Tilt series were collected at 200 kV in a Tecnai G 2 FEI microscope equipped with an ultrascan 1000 ccd camera (Gatan). Using Explore 3D, tilt series were collected with one-degree increments to cover the range between 65 and -65 degrees. We used Inspect 3D to align the collected images. The 3D reconstructions were computed using the SIRT with the Explore 3D software package (FEI). Image segmentation was performed using AMIRA® software.

### Statistical analysis

Mitochondria profile calculations were done for MUG-Chor1, U-CH2, and U2OS on 2D section using Image J software (Image Processing and Analysis in Java).

### Calcium imaging

The intracellular calcium concentration was assessed with the ratiometric fluorescent dye Fura-2-AM (Life Technologies, Carlsbad, CA). Cells were incubated for 1 h at 37°C under 5% CO_2_ in Tyrode solution (concentrations in mM: NaCl 137, glucose 5.6, KCl 5.4, NaHCO_3_ 2.2, MgCl_2_ 1.1, NaH_2_PO_4_, HEPES/Na 10, CaCl_2_ 1.8, pH 7.4) completed with 10 µM Fura-2-AM and 0.025% Pluronic F-127 (Sigma Aldrich, Vienna, Austria). Coverslips were washed to remove unloaded dye and placed in a Nikon fluorescence microscope. Fluorescence intensities were monitored with a cooled CCD camera at an emission wavelength of 510 nm, and the excitation was performed by a monochromator at 340 and 380 nm (VisiTech, Sunderland, UK). The data sampling rate was 1 ratio/s.

Histamin, ACh, 5-HT, and mes-ATP (all Sigma Aldrich, Vienna, Austria) at concentrations of 10 µM or 100 µM in Tyrode solution (as indicated), were applied by a superfusion system with a 7-channel perfusion pipette (List-electronic, Darmstadt, Germany). The system was driven by a valvebank (TSE, Bad Homburg, Germany) with a solution exchange time of less than 500 ms. Ca^2+^ free assay conditions were obtained by omitting Ca^2+^ in Tyrode's solution.

Stored images were analyzed using the QC 2000 software package (VisiTech), measured light intensity values of defined regions of interest were further evaluated with the Sigma Plot program (SPSS Inc., Erkrath, Germany). Background subtraction, rationing and calculation of the intracellular calcium concentration ([Ca^2+^]_i_) were performed offline.

### Identification and quantification of ceramide and glycosylceramide species

Lipid extraction was performed by a methyl tert-butyl ether extraction method according to Matyash *et al*. [26]. 1×10^6^ cells from MUG-Chor1, U-CH1, U2OS, and human skin fibroblasts were counted by CASY (Roche, Vienna, Austria). The methyl tert-butyl ether phase was transferred to a new vial and the solvent was removed under a nitrogen stream. Solvent-free lipid extracts were dissolved in 100 µl chloroform/methanol (CHCl_3_/MeOH) (1∶1 v/v) containing 250 pmol ceramide 12:0, ceramide 25:0, sphingomyelin 12:0, and GlyCer 12:0 each as internal standards. Sphingolipid species were first identified by Fourier transform ion cyclotron resonance mass spectrometry (LTQ-FT, Thermo Scientific, Waltham, MA) and subsequently quantified by triple quadruple LC-MS/MS (TSQ Quantum Ultra, Thermo Scientific) as described previously [27], [28]. Ceramide and GlyCer peak areas were calculated by QuanBrowser for all lipid species and quantification was performed by correlation to internal standards.

## Results

### Morphological features

Classical EM in combination with HPF and electron tomography enabled us to characterize the morphological difference between MUG-Chor1 cells, that exhibit the characteristic morphology of chordoma tissue.

**Figure 1:**
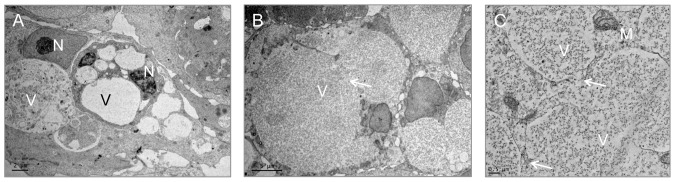
Conventional electron microscopy of vacuoles in chordoma cells (arrows indicate the reduced connection of the vacuoles; mitochondria (M); nucleolus (N); vacuoles (V)). A) MUG-Chor1 cells show a prominent nucleus, abundant vacuoles with a diverse density of material. B) Higher magnification of large densely filled vacuoles. C) Overview of single vacuoles with barely visible contact zones between the vacuoles.

**Figure 2:**
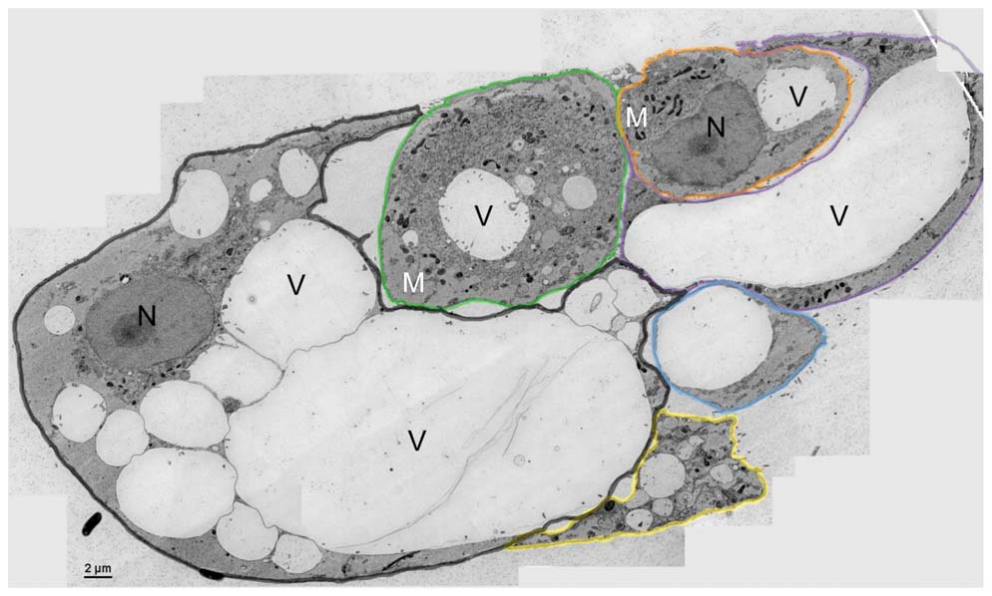
Electron microscopy overview of the chordoma cells (stitched image). HPF fixation was used to demonstrate the heterogeneity of chordoma cells. Cell 1 (black line) showed a prominent nucleus (N) and connected vacuoles (V) in different sizes. Cell 2 (green line) and cell 3 (orange line) present fewer vacuoles, abundant mitochondria (M), and dense ultrastructure of the cytoplasm. Cell 4 (violet line) and cell 5 (blue line) show prominent vacuoles surrounded by a small cytoplasm matrix, containing mitochondria. Cell 6 (yellow line) exhibit small vacuoles and less dense cytoplasma.

**Figure 3:**
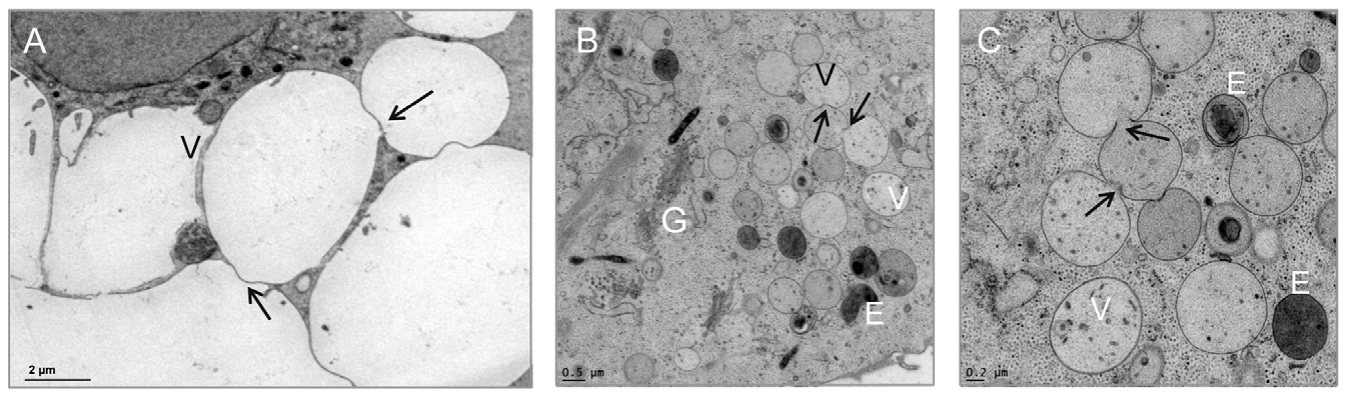
HPF fixed vacuoles within the chordoma cells. All micrographs indicate the connection of the vacuoles. A) Overview of the vacuoles (V); arrows indicate the network within the MUG-Chor1 cells. B) Cells show a high number of small vacuoles connected to each other as well as endosomes and Golgi apparatus, surrounded by dense cytoplasm. C) Higher magnification enables the visualization of the linkages between either small vacuoles or endosomes and vacuoles; arrows indicate the exchange of material within the vacuoles.

To visualize the ultrastructure of MUG-Chor1 cells we used chemical fixation (Figure 1) or HPF followed by freeze substitution (Figures 2, 3). Ultrastructural features of classically chemically fixed chordoma cells showed collapsed vacuoles filled either with or without undefined material of various electron densities. While the vacuoles appeared interconnected, their membranes seemed deformed, and therefore no clear conclusions could be drawn as to vacuole shape and content.

**Figure 4:**
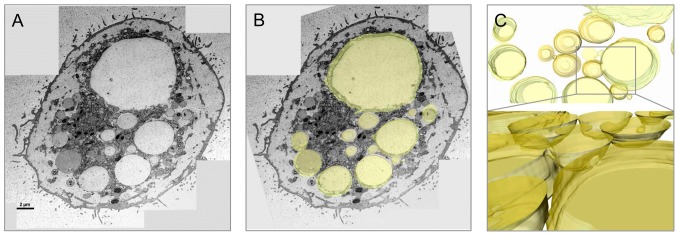
A three-dimensional reconstruction of 20 serial sections of high pressure frozen chordoma cells over a distance of 1.4 µm visualizes the connection of vacuoles. A) Top slice from the serial sections (70 nm). B and C) The surface model (yellow) of the vacuoles and the junctions of vacuoles within the cell.

In comparison to the chemical fixation technique, high pressure frozen, freeze substituted cells showed clearly defined, smooth, membranous structures and a better preserved morphology. This technology was able to clearly visualize the fine ultrastructural features of cell-to-cell interfaces as well as communicating organelles, as can be seen in the merged images in Figure 2 (intact vacuoles), Figure 3A and Figure 4 (communicating vacuoles). Because that HPF provides snap shots of the tissue frozen within milliseconds, it became apparent that an exchange of material occurred in vacuoles through openings in their membranes, thus indicating a communication between vacuoles. This observation is represented by a grey-shade gradient of one vacuole containing darker ingredients via a communication bridge to a subsequent vacuole containing a brighter content (Figure 3C: middle vacuole to top vacuole).

**Figure 5:**
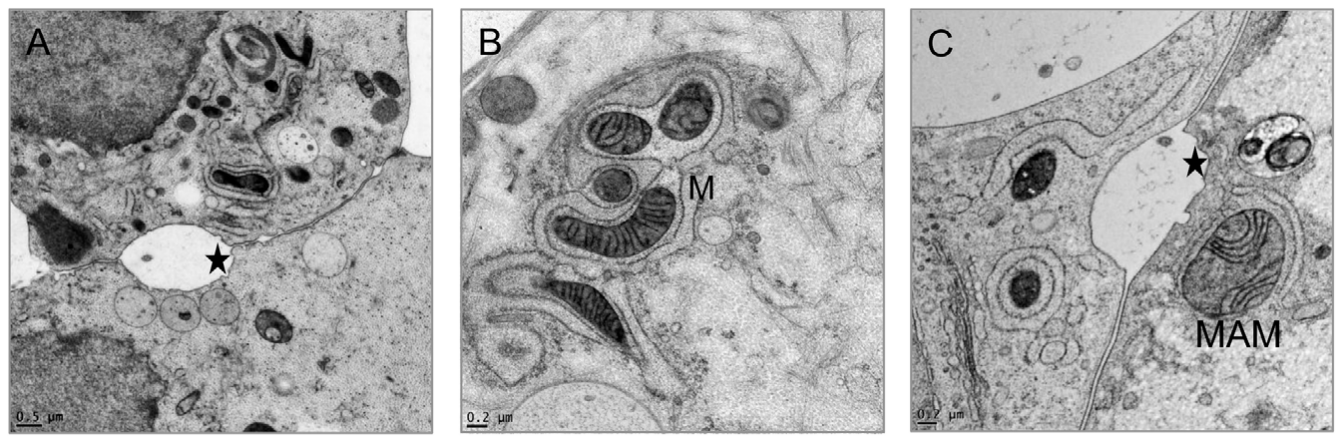
Structural association of mitochondrial membrane with the ER called MAM, represented by HPF. A) Abundant mitochondria within the cell, small connected vacuoles, and the connection of two chordoma cells with contact zones as well as excrescences (asterisk). B) Very close continuous contact zones of mitochondria (M) and ER. C) Connection of two chordoma cells with contact zones, excrescences (asterisk) and MAM complex.

**Figure 6:**
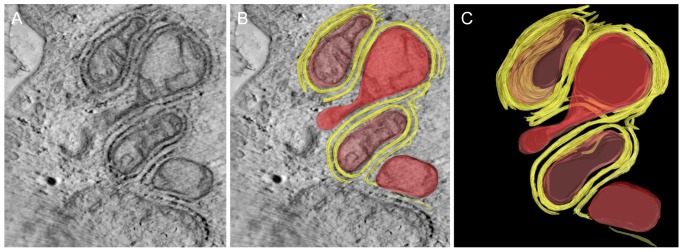
Electron tomogram showing ultrastructural details of mitochondria (M) embedded in several layers of endoplasmic reticulum (ER) (bar  =  0.5 µm). A) virtual image made from an electron tomogram showing how the mitochondria (M) are covered by ER. B) Virtual image with 3D contours of objects that were segmented (yellow: ER, red: M). C) 3D surface model of mitochondria covered by ER.

**Figure 7:**
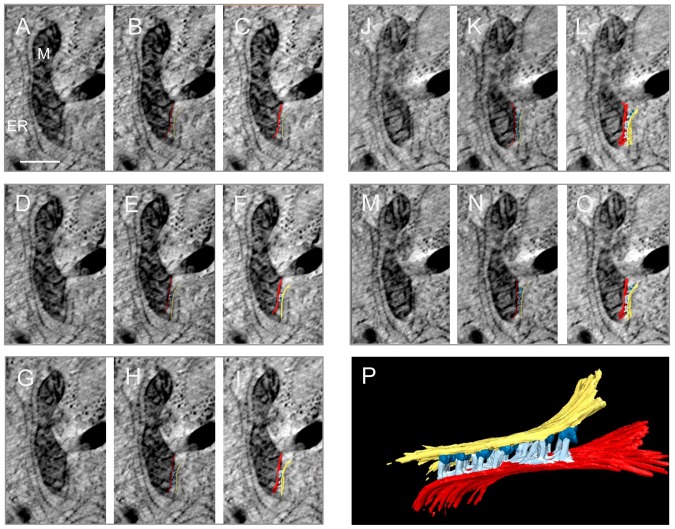
Electron tomogram showing ultrastructural details of the MAM complex (bar  =  0.5 µm). A, D, G, J, M) Sequence of virtual images made from an electron tomogram of the MAM complex. B, E, H, K, N) The same virtual images with the contours of the objects that were segmented. C, F, I, L, O) The same virtual images with the segmented 3D model shown as an overlay. P) Full reconstruction of the MAM complex showing that the mitochondria (M) and the endoplasmic reticulum (ER) are systematically connected via tethers as revealed in electron tomograms, mitochondria (outer mitochondria membrane red), endoplasmic reticulum (yellow), ribosomes (blue), and the tethers (light blue).

We implemented serial sections to visualize the communication in more detail and proved that 11 out of 15 vacuoles (70%) are linked via channels across 1.4 µm (Figure 4). A striking feature of the MUG-Chor1 and U-CH2 cells is their prominent close proximity between mitochondria and ER, known as the MAM complex. We were able to verify these features with both types of fixation, whereby again the HPF fixation created a better preserved ultrastructure compared to chemical fixation (Figures 1–7).

On many 2D sections the whole circumference of mitochondria was covered with ER (Figure 5 A–C). Measurements on mitochondrial profiles of MUG-Chor1 in a total area of 1,550.1 µm^2^ showed that 64.8% of the mitochondrial profiles appeared to be completely surrounded with ER, whereas in U-CH2 in an area of 1,588.4 µm^2^ 68.98% of mitochondrial profiles were fully encircled. In contrast, in the U2OS cells in an area of 1,571.4 µm^2^ none of the profiles were fully covered by ER (Table 1).

**Table 1.** Measurements on 2D sections on mitochondria profiles of MUG-Chor1, U-CH2, and U2OS cells.

Cell line	Measured area in µm^2^	Total number of mitchondria	Number of mitochondria fully surrounded by ER	Number of mitochondria partly surrounded by ER	Percentage % of mitochondria fully surrounded by ER
MUG-Chor 1	1,550.1	236	153	83	64.8
U-CH2	1,588.4	209	144	65	68.9
O2OS	1,571.4	193	0	193	0

To directly visualize the interaction between mitochondria and ER we used electron tomography, as it can reveal high resolution ultrastructural details. This showed how the mitochondria are embedded in several layers of ER. Only parts of the surface remained free (Figure 6). Sequences of virtual images were used to generate a 3D model of mitochondria, ER, ribosomes and the so called “tethers” that link the mitochondrial membranes to the MAM complex using Amira software. The tethers appeared to occur in a row of connections (Figure 7).

### Ca^2+^ release

**Figure 8:**
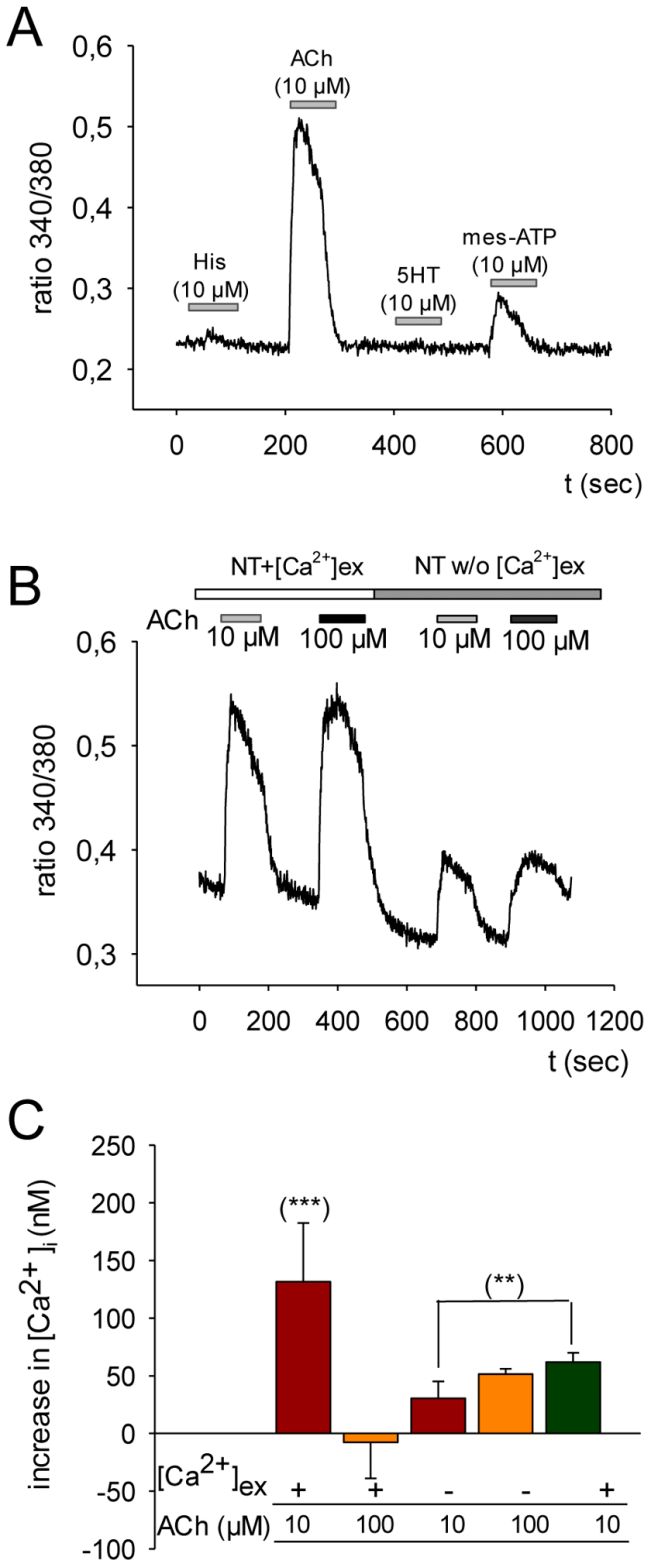
Intracellular calcium response to four different agonists in MUG-Chor1 cells was measured. A) Histamine (His), acetylcholine (ACh), serotonin (5HT), and P2Y purinoceptor agonist mes-ATP were applied at a concentration of 10 µM for a period of 60 seconds as indicated by the grey bars; the ratio between 340 nm and 380 nm is given. After stimulation, the respective agonist was washed out for another 60 seconds. B) The first two peaks of ratio values of MUG-Chor1 cells indicate ACh-application in the presence of extracellular calcium (NT+[Ca^2+^]_ex_; upper, open bar); peak reduction was induced by removing external calcium (NT w/o [Ca^2+^]_ex_; upper, grey bar). Small bars represent the time of perfusion with ACh at the two different concentrations (10 µM: lower, grey bar; 100 µM: lower, black bar). C) Within the bar chart a summary of ACh-induced changes is given. The first two bars represent effects in [Ca^2+^]_i_ induced by ACh (10 µM; 100 µM) given as average value (± standard deviation) of the change in intracellular calcium concentration [Ca^2+^]_i_ (nM). The usage of calcium within the perfusion solution as well as the different concentrations of ACh is indicated below the x-axis. Significant changes evaluated by the students t-test are given (***, p<0.001; **, p<0.01).

Changes in intracellular calcium [laCa^2+^]_i_ were analyzed by the application of the two neurotransmitters serotonin (5-HT) and ACh and furthermore by adding histamine or mes-ATP, respectively. As given within the representative original traces the addition of histamine and 5-HT did not induce any changes. On the other hand, we were able to demonstrate that particularly mes-ATP (less so at 130%), and ACh (to a greater extent with 220%), triggered a rise in [Ca^2+^]_i_ (Figure 8A). The use of two different concentrations in ACh (10 and 100 µM) did not alter the magnitude of the effect but the removal of external Ca^2+^ led to a reduction of measurable [Ca^2+^]_i_ by 58% (Figure 8B). Perfusion by ACh of MUG-Chor1 cells induced the intracellular Ca^2+^ within the nanomolar range by 131±50.9 nM, 100 µM ACh in comparison to 10 µM ACh did not further change its response (−7±31.2 nM). The observed reduction of [Ca^2+^]_i_ to 30±14.6 nM by Ca^2+^-free perfusion significantly changed to 61.8±8.1 nM when changing back to Ca^2+^-rich extracellular medium (Figure 8C).

### Lipid analysis

**Figure 9:**
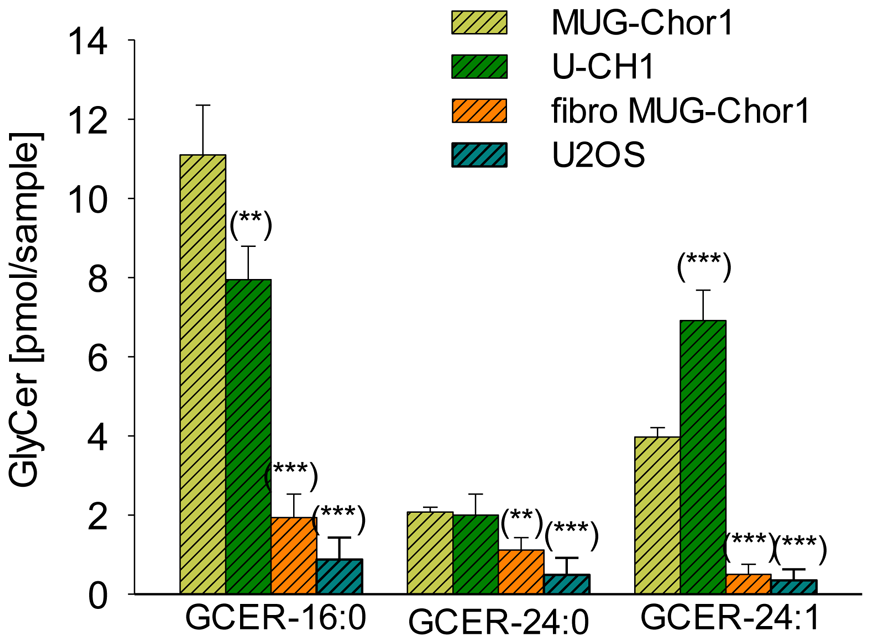
Lipid analyses. The GlyCer level (16:0; 24:0; 24:1) of chordoma cell lines (MUG-Chor1 and U-CH1) significantly increased compared to healthy human fibroblasts (fibro MUG-Chor1) and U2OS. The y-axis represents the amount of GlyCer (pg/mol). The x-axis represents the GlyCer with three different fatty acids attached to the amino group of sphingosine, namely palmitic acid (16: 0), lignoceric acid (24: 0), and nervonic acid (24: 1).

Since little is known about the lipid composition of chordomas, we performed a non-targeted differential lipidomic analysis. In this assay the level of GlyCer was strikingly and significantly different, while some triglyceride species (TG 52:1, TG 52:2, and TG 56:3) were marginally increased within chordoma cells (MUG-Chor1 and U-CH1) compared to osteosarcoma (U2OS) and human skin fibroblasts (data not shown). These lipid species were used for a concentrated targeted analysis, where the massive upregulation of GlyCer 16:0, GlyCer 24:0, and GlyCer 24:1 (Figure 9) could be corroborated. We discovered an accumulation of GlyCer in chordomas compared with fibroblasts and osteosarcoma. The slightly elevated triglyceride levels detected in the non-targeted lipidomic analysis could not be confirmed.

## Discussion

Chemotherapy plays a marginal role in the treatment of chordoma, with the current treatment options consisting of extensive surgical resection followed by high-dose radiation. The unique phenotype of chordoma – an accumulation of MAM complexes and chemoresistance prompted us to investigate ceramide and GlyCer levels as well as Ca^2+^ signaling in order to better understand the biology of chordoma cells.

Today, the quality of microscopic technology increased to very high level of specificity. Therefore, we have been able to paint a clear picture of the interaction of vacuoles and vesicles in one well-characterized chordoma cell line by maintaining the cellular structures.

Conventionally fixed vacuoles collapse because of fixation artefacts and therefore any existing network of vacuoles or communication interfaces of membranes is difficult to sustain. Hence we highly recommend HPF as the sample preparation of choice for electron microscopy to enable the imaging of biologically relevant structures avoiding artefacts and destruction. As early as 1968, Erlandson *et al*., showed cords and lobules in chordoma tissue and clusters of chordoma cells, as well as stellate and physaliferous populations of chordoma cells [10]. Due to having access to highly technical methods, we were able to more precisely observe the connection between vacuoles and visualize their interaction together with the bridges and the cell-cell interfaces.

It is well established that sub-compartiments of ER are in close spatial arrangement with mitochondria [29]. These lipid raft-like regions of ER are referred to as MAM and are, among other things, responsible for lipid syntheses, Ca^2+^ homeostasis regulation, and metabolism of glucose, phospholipids, and cholesterol [12], [30]–[32]. A striking feature of chordoma is how the association of mitochondria and ER does not seem to be organized into punctual continuous contact zones, as has been seen, for example, in liver cells [13]. The close proximity of mitochondria and ER could also be confirmed in an electron tomogram. The communication between ER and mitochondria is crucial for processes such as lipid synthesis/transport, assembly/secretion of lipoproteins and/or formation of lipid storage droplets as well as the regulation of Ca^2+^ homeostasis [32]–[34]. Szabadka *et al*. showed that in mammalian MAM, ER, and mitochondria are physically tethered to each other via the voltage-dependent anion-selective channel of the outer mitochondrial membrane and the inositol triphosphate (IP_3_) receptor from the ER responsible for Ca^2+^-release [35]. The molecular chaperone glucose-regulated protein 75 thereby identified as functioning as a communication link between these two ion channels and bearsthe responsibility for fatefulness and pathogenesis of the affected cells. Consequently the observed tight adjacency of ER and mitochondria equip cells with an efficient mitochondrial Ca^2+^ uptake as a consequence of IP_3_-induced Ca^2+^ release. Associated with cancer, Ca^2+^ signaling is described as being involved in each of the characteristics of cancer biology such as the ability to evade apoptosis, the capacity to metastasize and the promotion of angiogenesis, to name only a few [14]. Some cancers can already be associated with major changes in the expression of specific Ca^2+^ channels and pumps which lead to an increase or decrease of intracellular calcium and influence proliferation of the cells or apoptosis [14]. Specific investigations into the influx of Ca^2+^ as well as the function of intracellular Ca^2+^ release in tumor cells have identified Ca^2+^ as a good target for anticancer drug development [36].

The neurotransmitter ACh mainly acts on the peripheral and central nervous system by activating the inotropic nicotinic acetylcholine receptors (nAChR) and the metabotropic muscarinic acetylcholine receptors (mAChR). Both AChRs represent the central regulators of the neurotransmitter network and under activation increases intracellular cation concentrations that may trigger the direct stimulation of intracellular signaling cascades involved in regulation of cell proliferation, apoptosis, migration, and differentiation [37], [38].

Within our study we were able to identify functional AChRs by measuring changes in the intracellular Ca^2+^ concentration of FURA-2^AM^ loaded cells after ACh application. Interestingly, no such influence on intracellular Ca^2+^ signaling was detected by adding histamine or 5-HT, both agonists for activation of the IP_3_ pathway. A decent response to mesATP, attributable to the activation of one of the members of the P2X/P2Y purinergic receptors family was recorded. Interestingly, P2-receptors are said to represent an attractive target for the therapy of immuno-mediated disease and cancer [39], [40]. The reduction in measureable changes of [Ca^2+^]_i_ after ACh application by sequestering extracellular Ca^2+^ indicated, that changes were mainly carried by nAChR. The remaining increase might be due to expressed muscarinic receptor form implicating the expression and function of both ACh-receptors within the MUG-Chor1 cells.

Obviously the chordoma cell line MUG-Chor1 is adjustable by ACh, whereas a strong response in terms of changes in intracellular Ca^2+^ was detected. Changes in the composition of intracellular ions might lead to depolarization of the cell membrane and the further activation of Ca^2+^ channels.

To date nothing is known about the lipidome of chordomas. Due to the morphology of the tumor, the high number of vacuoles and the striking continuous contact zones of mitochondria and ER the lipid structure of chordoma cells may be important. Interestingly, we found a significantly higher amount of GlyCer in chordoma cells compared to healthy fibroblasts isolated from chordoma patients and osteosarcoma cells by using normal phase high-performance liquid chromatography coupled to atmospheric pressure chemical ionization-mass spectrometry. The accumulation of GlyCer concomitant with a poor response to chemotherapy features some multidrug-resistant cancer cells of breast, ovarian, colon, and epithelioid carcinomas [22]; [41]–[44]. The balance between UDP glucose ceramide glycosyltransferase (UGCG) and glycosidase beta acid (GBA) enzymes is responsible, among other things, for GlyCer. The former catalyzes the first glycosylation step in glycosphingolipid biosynthesis leading to GlyCer, the latter metabolizes GlyCer to glucose and ceramide. GlyCer is present in almost all eukaryotic organisms and plays a key role in the synthesis of hundreds of different glycosphingolipids. Glycosphingolipids are characteristic constituents of the plasma membrane and modulate cell proliferation as well as cell-cell interaction. Interestingly, the inhibition or down regulation of GlyCer production results in decreased drug resistance by increasing intracellular ceramide levels [44]. Lucci et al., demonstrated with a small cohort of tumor specimens that high GlyCer levels were associated with chemotherapy failure [45]. The high level of GlyCer in chordoma and the resulting low level of ceramides may cause intrinsic resistance to chemotherapy and may be anti-apoptotic and should be examined in more detail. Summarizing, the use of HPF instead of chemical fixation to characterize MUG-Chor1 chordoma cells by means of ultrastructure resulted in a significant improvement of morphological preservation. This, in turn, yielded ultrastructural details never before seen in chordoma cells; in particular a network of communicating vacuoles including defined structures at the sites of communication as well as a close spatial arrangement of ER and mitochondria, known as MAM complex. In addition to the new structural details, we found that GlyCer C16:0 and C24:1 increased. This finding might contribute to the drug resistance of chordoma cells as an accumulation of GlyCer was also reported to be responsible for drug resistance in other tumor entities. Whereas the significance of the communicating network of vacuoles is not yet clear, both the sensibility towards the AChR as well as the increased GlyCer represent promising links to new chordoma treatment.
